# Unexplained Bromide Toxicity Presenting as Hyperchloremia and a Negative Anion Gap

**DOI:** 10.7759/cureus.36218

**Published:** 2023-03-16

**Authors:** Sumugdha Rayamajhi, Shailendra Sharma, Hasan Iftikhar

**Affiliations:** 1 Internal Medicine, Michigan State University College of Human Medicine, East Lansing, USA; 2 Nephrology, Sparrow Health System, Lansing, USA; 3 Nephrology, Southside Kidney Specialists, Petersburg, USA

**Keywords:** altered mental state, electrolyte imbalance, hyperchloremia, negative anion gap, bromide toxicity

## Abstract

A high serum bromide level can cause erroneously high serum chloride levels measured through routine assays. Here, we describe a case of pseudohyperchloremia in which routine labs showed a negative anion gap and elevated chloride levels measured with ion-selective assay. The serum chloride level was found to be lower when measured with a chloridometer that employs a colorimetric method of quantification. The initial serum bromide level was elevated at 1100 mg/L that was confirmed by repeating the test that again showed an elevated level of 1600 mg/L and appeared to cause erroneous hyperchloremia when using conventional serum chloride quantification methods. Our case highlights lab errors and factitious hyperchloremia as a cause of the negative anion gap caused by bromism, even without a clear history of bromide exposure. The case also underscores the importance of chloride measurement using both colorimetric methods and ion-selective assay in the case of hyperchloremia.

## Introduction

Bromine is the third-lightest halogen. It is a deep red-colored liquid primarily used in the manufacturing of dyes, inks, flame retardants and other chemical agents like drilling oil and water treatment solutions [1]. Bromide concentration in serum is measured by x-ray fluorescence spectrometry, and the normal range is 3.2-5.6 mg/L [2]. Serum bromide concentration is difficult to quantify due to interference with other halogens. Significant environmental exposure mostly occurs in industrial settings through skin absorption, as seen in chemical plant workers in deep drilling, industries making flame retardants, to name a few [1]. Bromide toxicity however was clinically well recognized in the early 20th century when the use of bromide-containing drugs was widespread for a variety of ailments [1,3]. Toxic effects of bromide include neuropsychiatric disturbances, tremors, gait imbalance, rash, and dermatitis [4-7].

## Case presentation

An 82-year-old male presented to the emergency department with a sudden progressive decline in cognitive function, visual hallucinations, gait disturbance and multiple falls over the last two weeks. His medical history was significant for squamous cell cancer of the head and neck (which was treated with radiation and required percutaneous endoscopic gastrostomy tube placement), severe scoliosis, pulmonary fibrosis and myasthenia gravis. He was on intravenous immunoglobulin infusion every four weeks and an ipratropium bromide 20 mcg inhaler two to three times daily. Notably, he was not on pyridostigmine bromide either at the time of presentation or in the past. Physical examination was significant for blood pressure of 93/55 mm Hg, and other vitals were within normal limits. He had fluctuating mental status but had an otherwise normal neurologic exam (except gait that could not be assessed due to patient's profound weakness).

Initial laboratory tests revealed a normal complete blood count, liver function tests, urinalysis and blood gas. His TSH, salicylate, acetaminophen, B12 and cortisol levels were also unremarkable. Serum chemistries revealed elevated serum chloride levels of 163 mmol/L and 175 mmol/L (repeat) with a calculated anion gap of negative 65 on presentation. Head CT showed no acute pathology while MRI could not be performed due to severe scoliosis. Multiple repeat labs continued to show high chloride concentrations of 170, >175, 170, 174, 167 and >175 mmol/L. Later, simultaneous chloride measurements were obtained by employing indirect ion-selective electrode, or ISE (Siemens Vista 1500; Siemens Healthcare Diagnostics) and the colorimetric method, which showed values of 135 and 103 mmol/L, respectively. Urine chloride values using both methods were 61 and 45 mmol/L, respectively, which were normal. A chloridometer employing the colorimetric method is less susceptible to interference from other halide ions like bromide, which explains the discordance between the two methods. Concurrent serum bromide levels were reported as 1100 and 1600 mg/L on repeat measurement. Patient’s family and power of attorney refused both saline diuresis and hemodialysis for bromide clearance. The patient expired at his home six weeks following inpatient discharge.

## Discussion

The incidence of bromide toxicity (bromism) has declined precipitously following a sharp drop in the use of bromide-containing drugs since the early 20th century [3]. Bromide was used in medicinal drugs for indications as broad as insomnia, hysteria, anxiety, and even excessive libido, making it one of the most frequently used class of medicinal drugs [2,3]. Bromide intoxication was not an uncommon reason for psychiatric admission in that era. Up to 8% of those patients were found to have elevated serum bromide levels [3].

Typical presentation of bromism includes agitation, emotional lability, weakness, slurred speech and gait disturbances [3]. More serious central nervous system (CNS) disturbances including coma and death can be induced by very high bromide levels [6,8]. Additionally, hypersensitivity to bromides manifests as bromoderma, with characteristic histologic evidence of bromide salt deposition resulting in inflammation and injury [7]. Normal human serum was found to contain a mean level of 252 microgram bromide per 100 mL, with a range of 200 ± 42 microgram/100 mL [9]. A bromide blood concentration of >1000 microgram/100 mL can cause severe toxicity [10].

Measured chloride values as high as 170 mmol/L (normal 96-106 mmol/L) are seen with rising serum bromide levels. This discrepancy is a result of bromide ions reacting with most analytic reagents more strongly than native chloride. In equilibrium, bromide will displace a portion of chloride ions to reach a steady-state concentration, which theoretically should not alter the total halide concentration. However, when both chloride and bromide ions react with the reagent, the minority bromide ions carry more weight due to stronger affinity for the reagent. This is most apparent with an ion-selective method for determining serum chloride. Nevertheless, other methods of measuring chloride vis-à-vis colorimetric methods and coulometry are susceptible as well [2]. The ion-selective method is more widely used in clinical laboratories, including at our center where we use inductively coupled plasma mass spectrometry, or ICP/MS (Labcorp, Inc., Burlington, NC), and 50 mg/L is used as a reporting limit.

Another caveat to measuring bromide levels is possible interference with iodine, as it is present in contrast agents. It is recommended by the testing laboratory that serum bromide measurement be delayed to at least 96 hours after contrast administration [5]. While we detected an aberrantly high value of serum iodide on initial measurement following a CT scan with contrast, serum bromide concentration was confirmed by repeat measurements and was consistent with known clinical presentation.

Several available drugs are formulated as bromide salts (Table 1) [11]. Bromide is not a molecular component of any drug in use today except for bromovalerylurea, which is a weak hypnotic and anti-inflammatory drug available in East Asia [6,9].** **Bromide is absorbed from the gut in trace amounts. It has a slow rate of elimination with a half-life of 12 days and a ‘normal’ steady-state concentration of 2.38 mg/L [7]. Exposure must be prolonged to achieve a higher steady-state concentration. Bromide is metabolized by liver and at the tissue site by cholinesterase and excreted renally (80%-90% of the drug is excreted unchanged). Reported treatment options include saline diuresis with a loop diuretic, which has been shown to reduce the half-life of bromide to less than three days [12].** **Hemodialysis has been shown to hasten resolution to a few hours [13].

**Table 1:** Drugs formulated as bromide salts

Drug name	Drug category	Bromide content	Typical dose	Absorption/bioavailability	Estimated bromide exposure
Ipratropium bromide	Antimuscarinic	250 mg	Up to 200 mcg/day	7%	3.5 mcg/day
Tiotropium bromide	Antimuscarinic	170 mg	2.5 mcg/day	Minimal	<0.2 mcg/day
Methscopolamine bromide	Antimuscarinic	200 mg	30 mcg/day	10%-25%	1 mg/day
Pyridogstigmine bromide	Anticholinesterase	310 mg	600 mcg/day	10%-20%	15-30 mg/day
Neostigmine bromide	Anticholinesterase	260 mg	PO 150 mcg/day, parenteral 0.5-2.5 mg	1%-2%	4-8 mg/day, parenteral 0.2-1 mg/day
Vecuronium bromide	Neuromuscular blocker	130 mg	7-10 mg IV	100%	1-1.3 mg/dose
Rocuronium bromide	Neuromuscular blocker	130 mg	0.6-1 mg/kg followed by 8-12 mcg/kg/min	100%	50-80 mg/12 hour infusion
Dextromethorphan hydrobromide	Antitussive	230 mg	120 mg/day	Rapidly and well absorbed	28 mg/day
Halothane	Inhaled anesthetic	410 mg	Variable	Partition coefficient 2.4	Variable
Methylnatrexone bromide	Opiate receptor antagonist	180 mg	PO 450 mg/day, parenteral 12 mL/day	Not established	2 mg/day for parenteral dose
Bromvalerylurea	Sedative	360 mg	800-2400 mg/day	Not established	50-150 mg/day
Pipobroman	Chemotherapeutic agent	450 mg	75 mg/day	Not established	34 mg/day

Table 1 emphasizes that the extent of bromide exposure due to a drug would be determined by the relative content of bromide in a typical dose as well as a drug’s bioavailability. It is noteworthy that ipratropium bromide is usually administered in minute doses (up to 200 mcg/day) and has minimal systemic absorption. In fact, even ingesting an entire bottle of ipratropium bromide will only deliver less than 1 mg of bromide. This contrasts with pyridostigmine bromide that is usually dosed at up to 600 mg/day and hence has substantially a higher bromide content, delivering a dose of about 15-30 mg of bromide/day.

An elevated bromide level of 1100 mg/L was confirmed in our patient with a repeat measurement of 1600 mg/L. The validity of the test measuring serum electrolytes was confirmed with the lab and there was no evidence of systematic errors in calibration. No other case of abnormally high serum chloride was reported in the hospital during the same period.

Sangster et al. investigated effects of various doses of ingested bromide to serum levels [14]. In this study, 0, 4 and 9 mg/kg/day of bromide was administered to 14 healthy volunteers for 12 weeks in a double-blind fashion. Resulting serum concentrations were 5.6-6.3, 170-240 and 340-390 mg/L for the three dosing groups, respectively. Even relatively high doses of ingested bromide over 12 weeks failed to produce levels in the toxic range in this small study. The pharmacokinetics of bromide ion are not be fully understood; it is still meaningful to recognize that a negative anion gap with a compatible clinical picture should be presumed as bromide toxicity and treated immediately and appropriately. Due to the rare incidence of bromism, it is important to provide additional counseling for patients and families to understand the condition's impact and treatment options to support better patient outcomes.

During our literature review, we could find minimal relevant biomedical research on bromide toxicity; rarity of this could be due to under-reporting and under-recognition. It is important to continue adding to the limited literature and research so that healthcare providers can have awareness of bromide toxicity. High suspicion is necessary especially with a history of bromide exposure and metabolic abnormalities as mentioned above in our case.

## Conclusions

This case report provides several important clinically relevant clinical teaching points. A very high index of suspicion is required to diagnose bromide toxicity. Similar properties such as other halogens and high bromide levels can masquerade as hyperchloremia with a large negative anion gap. Finding hyperchloremia and a high negative anion gap should prompt providers to review medications that may have bromide in them and repeat chloride measurement through a traditional chloridometer using the colorimetric technique. This case provides beneficial material for future research to explore further possibilities relating to bromide toxicity.
